# Immunogenicity of glycosylphosphatidylinositol-anchored micronemal antigen in natural *Plasmodium vivax* exposure

**DOI:** 10.1186/s12936-017-1967-9

**Published:** 2017-08-22

**Authors:** Siriruk Changrob, Jin-Hee Han, Kwon-Soo Ha, Won Sun Park, Seok-Ho Hong, Patchanee Chootong, Eun-Taek Han

**Affiliations:** 1 Department of Clinical Microbiology and Applied Technology, Faculty of Medical Technology, Mahidol University, Bangkok, 10700 Thailand; 2 Department of Medical Environmental Biology and Tropical Medicine, School of Medicine, Kangwon National University, Chuncheon, Gangwon-do 200-701 Republic of Korea; 3 Department of Cellular and Molecular Biology, School of Medicine, Kangwon National University, Chuncheon, Gangwon-do 200-701 Republic of Korea; 4 Department of Physiology, School of Medicine, Kangwon National University, Chuncheon, Gangwon-do 200-701 Republic of Korea; 5 Department of Internal Medicine, School of Medicine, Kangwon National University, Chuncheon, Gangwon-do 200-701 Republic of Korea

**Keywords:** Immunogenicity, *Plasmodium vivax*, GAMA

## Abstract

**Background:**

*Plasmodium vivax* is the most geographically widespread malaria species and codominates with *Plasmodium falciparum*, the deadliest form of the malaria parasite. For the last few years, the number of vivax malaria cases has increased, but vivax malaria is still considered a neglected disease. During the blood stages of their life cycle, *P. vivax* parasites export several hundred proteins into host red blood cells. Some of these exported proteins have been discovered and studied for use as a blood-stage malaria vaccine. The *P. vivax* glycosylphosphatidylinositol (GPI)-anchored micronemal antigen (PvGAMA) was identified in previous study, which plays an important role in parasite invasion. To support the hypothesis that PvGAMA can induce an immune response in natural exposure, the antibody responses and cellular immunity against this antigen was demonstrated during and post-infection.

**Methods:**

The recombinant protein PvGAMA was expressed and purified by wheat germ cell-free (WGCF) system. The analysis of humoral and cellular immune responses to the PvGAMA antigen during infection and post-infection with the *P. vivax* parasite were done by enzyme-linked immunosorbent assay (ELISA) techniques.

**Results:**

During *P. vivax* infection, 95% of patients showed significant antibody responses to PvGAMA antigen. The cytophilic IgG1 and IgG3 isotypes were the major isotypes produced in response to PvGAMA. A cross-sectional study of anti-PvGAMA responses during and post-infection with *P. vivax* found that the majority of individuals, approximately 54% of patients, were shown to maintain a positive anti-PvGAMA titre at 3 months post-infection, and some patients had the ability to maintain an antibody response for up to 12 months post-infection. Moreover, PvGAMA had the ability to stimulate a cellular immune response that was characterized by the production of the cytokines IL-2, IFN-γ and IL-10. The levels of the cytokines IFN-γ and IL-10 were significantly increased in PvGAMA-stimulated lymphocyte cultures.

**Conclusions:**

Taken together, PvGAMA had potential to induce an immune response both humoral and cellular immunity in naturally acquired *P. vivax* infection individuals during infection and post-infection. Therefore, PvGAMA could be as a vaccine candidate to stimulate immune response against *P. vivax* infection.

## Background

Malaria remains one of the most devastating infectious diseases, killing approximately 429,000 people in 2015. *Plasmodium vivax* is the most geographically widespread malaria parasite species and codominates with *Plasmodium falciparum* as a cause of human malaria. *Plasmodium vivax* has previously been considered benign; however, during the last few years, *P. vivax* is estimated to have been responsible for 3100 deaths [1]. Due to some specific characteristics that make it distinct from *P. falciparum,* such as the fact that the *P. vivax* hypnozoite can become reactivated, cause repeated clinical malaria attacks, and undergo onward transmission, emerging data indicate that *P. vivax* is very often associated with severe symptoms [2, 3]. Developing a vaccine is considered to be one of several strategies to control and eradicate vivax malaria. A greater understanding and improved knowledge of protective immunity against the parasite are required to achieve this goal.

One approach to *P. vivax* vaccine development is to focus on the interruption of merozoite invasion of erythrocytes via the critical ligand-receptor interaction between Duffy-binding protein (DBP) and its corresponding surface receptor on erythrocytes: namely, Duffy antigen receptor for chemokine (DARC). PvDBP is a leading target for a *P. vivax* vaccine candidate since this antigen could induce an antibody response that prevented *P. vivax* binding to erythrocytes and parasite invasion [4–8]. However, the description of cases of vivax malaria among Duffy-negative individuals was reported in sub-Saharan Africans [9]. It is suggested that alternative ligands and pathways play a role in reticulocyte invasion. As such, finding additional vaccine candidates is necessary for controlling malaria.

The glycosylphosphatidylinositol-anchored proteins (GPI-APs) are one type of protein antigen important for parasite invasion [10]. Several GPI-APs have been discovered and characterized as blood-stage vaccine candidates against *P. vivax* infection; these include PvMSP-1, PvMSP-3, PvMSP-4, PvMSP-8, PvMSP-9, PvMSP-10, Pv12, Pv34, Pv38 and PvMSP1P [3, 11]. A new erythrocyte-binding protein named GPI-anchored micronemal antigen (GAMA) was first identified in *P. falciparum* [12]. The importance of conformation-specific epitopes was revealed by a previous study showing that only an antibody against PfGAMA expressed by the wheat germ cell-free (WGCF) system can inhibit *P. falciparum* invasion; by contrast, antibodies generated from PfGAMA expressed by *Escherichia coli* failed to inhibit invasion [12]. The ortholog of PfGAMA, *P. vivax* GPI-anchored micronemal antigen (PvGAMA), has been successfully produced and its function was characterized in previous study [13]. PvGAMA was localized at the microneme in the mature schizont blood stage. This antigen can bind to human erythrocytes regardless of the Duffy antigen status. Importantly, the antibodies against the PvGAMA fragment inhibited erythrocyte binding, suggesting that this protein is a novel blood-stage vaccine candidate [13]. Thus, a greater understanding of PvGAMA antigenicity and immunogenicity in the induction of the immune response, as well as in the longevity of the antibody response, is required. In this study, the immunogenicity of PvGAMA in the induction of the immune response was demonstrated, both for antibody and cellular responses during and post-infection with *P. vivax*.

## Methods

### Sample collection

To study humoral immune responses to PvGAMA antigen, a study was carried out in an area of low malaria transmission, Chumphon Province, in the southern part of Thailand, where both *P. vivax* and *P. falciparum* commonly occur. Ten mL samples of heparinized peripheral blood were collected from individuals acutely infected with *P. vivax* (n = 40), and from those who had been in recovery for 3 months (n = 35), 9 months (n = 15) and 12 months (n = 14) between May 2014 and May 2015. The age of subjects ranged from 18 to 63 years old. Fifteen samples from villagers who lived in malaria-endemic areas for more than 5 years and showed a negative parasitaemia smear at the time of collection were obtained. Giemsa staining of thick and thin peripheral blood films was used for *P. vivax* diagnosis. The density of malaria parasites in the blood of infected patients ranges from 200 parasite/μL to 15,000 of parasites/μL. The nested PCR was used to confirm *P. vivax* infection during acute phase and to confirm the presence of parasite at 3, 9 and 12 months post-infection.

To study the cellular immune responses to PvGAMA, peripheral blood mononuclear cell (PBMC) samples were obtained from acutely infected individuals (n = 11) and individuals who had been in recovery for 8–10 weeks (n = 11). Blood samples were taken from healthy persons (n = 50) who had never been exposed to malaria, which were held at the Faculty of Medical Technology, Mahidol University, were used as the naive controls in this study. The study was approved by the committee on Human Rights Related to Human Experimentation, Mahidol University, Thailand (MUIRB2012/079.2408).

### Nested PCR detection of *P. vivax* parasite

The nested PCR was performed as described previously [14]. In briefly, genomic DNA was extracted from blood samples, 2 µL of DNA template in a 20 µL reaction mixture was amplified by primer rPLU1_F (TCA AAG ATT AAG CCA TGC AAG TGA) and primer rPLU5_R (CCT GTT GTT GCC TTA AAC TTC). The reactions were as follows: initial denaturation, 95 °C for 5 min; denaturation, 95 °C for 30 s; annealing, 55 °C for 1 min; extension, 72 °C for 2 min. Then, the secondary PCR was done by using product from first reaction as DNA template. The DNA template was mix with same condition of 20 µL reaction mixture and amplified by *P. vivax* species-specific nested PCR primers rVIV1_F (CGC TTC TAG CTT AAT CCA CAT AAC TGA TAC) and primer VIV1_R (ACT TCC AAG CCG AAG CAA AGA AAG TCC TTA). The secondary PCR reactions were as follows: initial denaturation, 95 °C for 5 min; denaturation, 95 °C for 30 s; annealing, 60 °C for 30 s; extension, 72 °C for 1 min. The PCR product was observed in 1.5% agarose gel stained with fluorescent nucleic acid gel stains.

### Recombinant PvGAMA proteins

The recombinant protein PvGAMA-Ecto domain comprising amino acid 22–771 was expressed and purified as previously described [13]. Briefly, the PVX_088910 fragments encoding PvGAMA-ectodomain and a hexahistidine (His) tag at the C-terminus, were amplified using sense primers with *Xho*I sites and antisense primers with *Bam*HI restriction sites. The amplified fragments were then restricted and ligated into the WGCF expression vector pEU-E01-His-TEV-MCS (CellFree Sciences, Matsuyama, Japan). The cloned inserts were sequenced using an ABI 3700 Genetic Analyzer (Genotech, Daejeon, Korea). The recombinant proteins with His tags were expressed using a WGCF system (CellFree Sciences) and purified using a Nickel-Sepharose column (GE Healthcare Life Sciences, Uppsala, Sweden).

### IgG antibody responses against PvGAMA

The levels of antibody produced in response to PvGAMA in individuals with *P. vivax* infection were measured by a conventional enzyme-linked immunosorbent assay (ELISA). In brief, 5 μg/mL of recombinant PvGAMA protein was coated overnight in 96-well microtitre plates. Next, blocking buffer, 5% skimmed milk, was added to each well and incubated for 2 h. After washing the plates 3 times, human plasma diluted 1:200 with blocking buffer was added to the coated plates and incubated for 1 h. The wells were extensively washed, followed by the addition of 1:1000-diluted alkaline phosphatase-conjugated goat anti-human IgG antibodies into each well and incubated for 1 h. The plate was washed five times before adding ABTS [2,2′-azino-bis(3-ethylbenzothiazoline-6-sulphonic acid)] substrate into the wells, and the plate was left for 60 min in the dark to allow the colorimetric reaction to occur. The optical density of each of the sample wells was measured at 405 nm (OD_405_) using a Synergy™ HTX Multi-Mode Microplate Reader (BioTek, Winooski, VT, USA). Samples from all subjects were analysed in duplicate, and mean values were used in the subsequent analyses.

### IgG isotype responses to PvGAMA

The prevalence of the IgG isotype response specific for PvGAMA was demonstrated in comparison to PvDBPII since PvDBPII is a leading blood-stage vaccine candidate against *P. vivax* (PvDBPII was a gift from John H. Adams, University of South Florida, FL, USA). Plasma samples from patients who were acutely infected with *P. vivax* (n = 29) and from healthy controls (n = 18) were analysed using an ELISA assay. Briefly, 5 μg/mL of rPvGAMA or 2 μg/mL of rPvDBPII was immobilized on 96-well microtitre plates by incubation at 4 °C overnight. Non-specific protein binding was blocked using a blocking buffer containing 5% skimmed milk for 2 h. The plates were incubated with 100 μL of plasma at a dilution of 1:100 in blocking buffer. Horseradish peroxidase-conjugated anti-human IgG1, IgG2, IgG3, and IgG4 antibodies diluted 1:1000 in blocking buffer were used for detection. The optical density of the reaction was measured at 450 nm after the addition of the enzyme–substrate (TMB). The cut-off value was obtained from the mean plus two standard deviations (SDs) of the OD of all of the healthy control plasma samples.

### PBMC stimulation assay

A lymphocyte proliferation assay (LPA) was carried out to evaluate cellular immunity against PvGAMA. First, PBMCs were isolated from patients who were acutely infected with *P. vivax* and those who had been in recovery for 8–10 weeks using Ficoll-Hypaque (Stemcell Technologies, Vancouver, Canada). PBMCs were then washed twice with incomplete Roswell Park Memorial Institute (RPMI) 1640 medium. PBMCs were used for the LPA when viability was greater than 90%. PBMCs were suspended in complete RPMI 1640 medium containing 10% fetal bovine serum (FBS), and 2.5  ×  10^5^ PBMCs were added into each well of a 96-well flat-bottomed tissue culture plate (Corning Inc., New York, NY, USA). The cells were then stimulated by 10 μg/mL of purified rPvGAMA or rPvDBPII. The complete RPMI 1640 medium and PBMCs stimulated with 2% v/v of phytohaemagglutinin (PHA) were used as negative and positive controls, respectively. Cells were cultured for 96 h at 37 °C under 5% CO_2_. After 96 h, the culture supernatant was harvested for cytokine detection.

### Cytokine assay

The cytokine levels in human lymphocyte culture supernatants were detected after 96 h of stimulation with PvGAMA using an ELISA Cytokine Kit (BD OptEIA™; BD Biosciences, San Diego, CA, USA), as described previously [15]. Briefly, 100 μL of culture supernatant was added into wells pre-coated with monoclonal antibodies specific to IL-2, IL-10 or IFN-γ. After 2 h of incubation at room temperature, streptavidin–horseradish peroxidase conjugate mixed with biotinylated anti-human cytokine antibodies was added, and then incubated for 1 h. The colorimetric reaction was developed by the addition of 100 μL of TMB substrate solution and the OD was measured at 450 nm (OD_450_) within 30 min after the addition of a stop solution. A human cytokine standard curve was used as the experimental control to calculate cytokine concentrations. All experiments were done in duplicate, and the means of the two values were used for subsequent analyses.

### Statistical analysis

Data and graphs were analysed using the GraphPad Prism software (San Diego, CA, USA). A nonparametric Mann–Whitney *t* test was used to determine where there was a significant difference between the study groups in terms of the levels of antibody against PvGAMA. A Wilcoxon matched-pairs signed-rank test was used to evaluate the significance of cellular responses to PvGAMA or PvDBPII in each individual. In all analyses, a *P* value less than 0.05 (*P* <  0.05) was deemed statistically significant.

## Results

### Naturally acquired IgG responses to PvGAMA

**Fig. 1:**
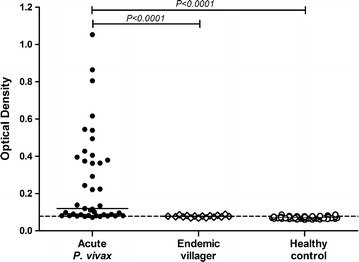
Humoral immune response to the PvGAMA antigen. The total levels of IgG specific to recombinant PvGAMA in human plasma from patients with acute *P. vivax* infection patients (n = 40), villagers in malaria endemic areas (n = 15) and healthy controls (n = 50) were measured by conventional ELISA. *Each symbol* represents the antibody titre of one individual. The *solid line* shows the median antibody titre in each group. The *dotted line* indicates the cut-off values

**Fig. 2:**
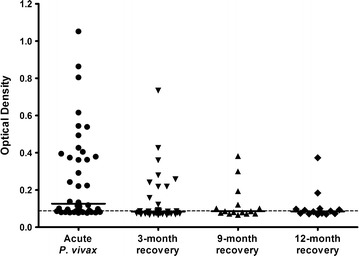
The antigenicity of PvGAMA during and post-infection with *Plasmodium vivax*. The anti-PvGAMA responses were measured in acutely infected individuals (n = 40) and in patients who had been in recovery for 3 months (n = 35), 9 months (n = 15) and 12 months (n = 14) by conventional ELISA assay. *Each symbol* represents the antibody titre of one individual. The *solid line* shows the median antibody titre in each group. The *dotted line* indicates the cut-off values

To evaluate the humoral immune response against PvGAMA in sera from Thai individuals, the antibody response to this antigen was examined by indirect ELISA. The results showed a significant increase in the anti-PvGAMA antibody response in patients who were acutely infected with *P. vivax* compared with the immune villagers (*P* < 0.0001; acute *P. vivax* OD value = 0.266 ± 0.246; endemic villagers OD value = 0.078 ± 0.006) and healthy controls (*P* < 0.0001; acute *P. vivax* OD value = 0.266 ± 0.246; healthy control OD value = 0.071 ± 0.004) (Fig. 1). There was no PvGAMA-seropositive individual found in the group of endemic villagers (Fig. 1). The seroprevalence of anti-PvGAMA response were 95.0, 54.3, 66.7 and 57.1% during acute infection, at 3, 9 and 12 months post-infection, respectively (Fig. 2; Table 1).

**Table 1:** The serological analysis of anti-PvGAMA responses during acute *Plasmodium vivax* infection and post-infection

Subjects	% Positive prevalence^a^	Optical density (OD)				
		Min.	Max.	Median	SD	*P* value^b^
Acute phase	95.0	0.077	1.052	0.126	0.246	<0.0001
3 months of recovery	54.3	0.065	0.734	0.084	0.137	<0.0001
9 months of recovery	66.7	0.061	0.382	0.081	0.067	<0.0001
12 months of recovery	57.1	0.068	0.373	0.085	0.081	0.0004

*Min.* the lowest antibody titre of each group of subjects, *Max.* the highest antibody titre of each group of subjects, *Median* the median of antibody titre presented as the median OD value of each group of subjects, *SD* the standard deviation of antibody titre of each group of subjects, *NS* not significant

^a^Positive prevalence: the percentage of seropositive individuals who had OD values greater than the cut-off value (mean ± 2 SD of the OD value of healthy controls)

^b^The *P* value of the difference between the mean antibody titre of *P. vivax*-infected subjects and those in the recovery phase or healthy controls, as calculated using the Mann–Whitney *U* test

### The longevity of anti-PvGAMA antibody responses in *P. vivax* infection

**Fig. 3:**
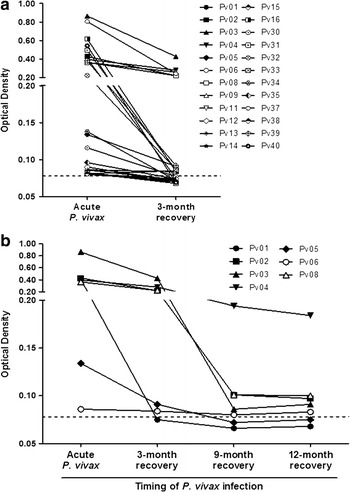
The kinetics of anti-PvGAMA responses in acute infection and after anti-malarial treatment. Only individuals who were seropositive to PvGAMA in the acute phase were part of subsequent experiments involving the detection of antibody responses. **a** A comparison of the anti-PvGAMA antibody levels in individuals during acute infection and at 3 months post-infection (n = 24). **b** Antibody responses to PvGAMA in PvGAMA-seropositive patients (n = 7) and after 3, 9 and 12 months of recovery

To observe the longevity of anti-PvGAMA responses after anti-malarial treatment, a cohort study was conducted to monitoring of antibody responses in individuals during and at 3, 9 and 12 months post-infection. After 3 months infection, 13 individuals from 24 infected patients maintained a positive of anti-PvGAMA response whereas 11 patients, PV01, PV11, PV12, PV13, PV14, PV30, PV33, PV34, PV35, PV38 and PV40, became seronegative (Fig. 3a; Table 2).

**Table 2:** The longitudinal analysis of anti-PvGAMA responses in individuals during acute *Plasmodium vivax* infection and 3 months post-infection

Subject	Optical density (OD)	
	Acute phase	3 months post-infection
Pv01	0.405	0.075
Pv02	0.427	0.218
Pv03	0.864	0.426
Pv04	0.395	0.279
Pv05	0.134	0.091
Pv06	0.086	0.084
Pv08	0.363	0.219
Pv09	0.362	0.258
Pv11	0.081	0.074
Pv12	0.090	0.071
Pv13	0.086	0.073
Pv14	0.080	0.072
Pv15	0.081	0.085
Pv16	0.616	0.084
Pv30	0.116	0.077
Pv31	0.494	0.086
Pv32	0.221	0.087
Pv33	0.082	0.068
Pv34	0.138	0.072
Pv35	0.096	0.074
Pv37	0.805	0.241
Pv38	0.081	0.070
Pv39	0.379	0.092
Pv40	0.544	0.069

Seropositive: the individuals who had OD values greater than or equal to the cut-off value (mean ± 2 SD of the OD value of healthy controls = 0.078)

Additionally, the further analysis of stable anti-PvGAMA responses by following the antibody titre in individuals (n = 7) until 12 months post-infection showed that 6 of 7 individuals had a positive anti-PvGAMA responses after 3 months of infection. At 9 months post-infection, the patients PV02, PV03, PV04, PV06 and PV08 maintained a seropositive level of anti-PvGAMA titre, whereas the patients PV01 and PV05 were seronegative (Fig. 3b). Interestingly, the monitoring of anti-PvGAMA responses at 12 months post-infection showed that those patients who were positive for anti-PvGAMA antibody at 9 months maintained their antibody responses (Fig. 3b).

### Predominant IgG1 and IgG3 isotype responses to PvGAMA

**Fig. 4:**
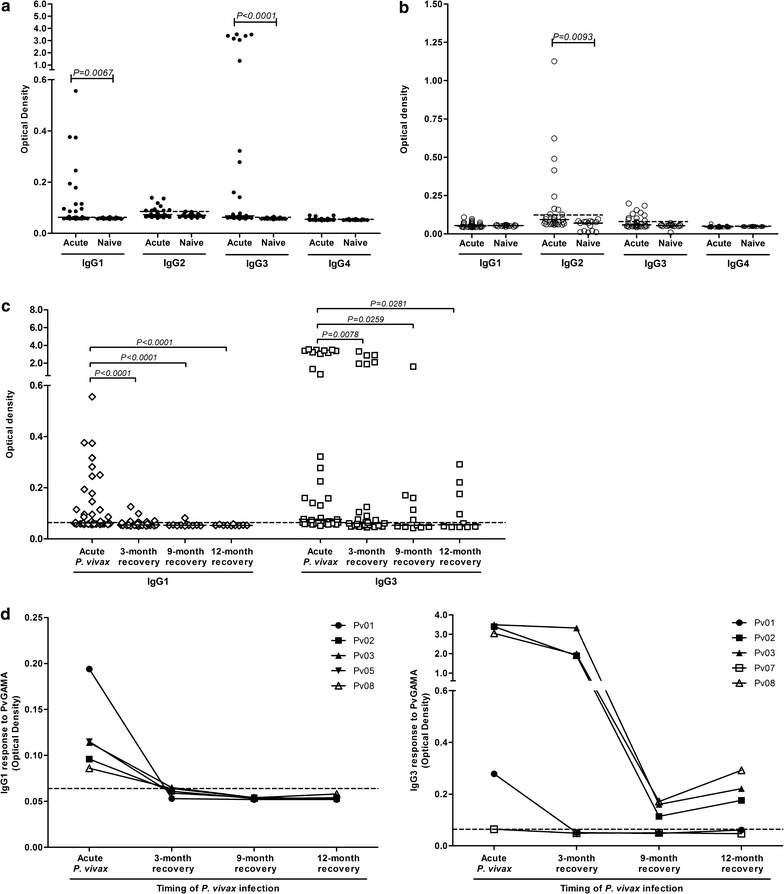
IgG isotype responses to the PvGAMA antigen. The prevalence of the IgG isotype response to PvGAMA (**a**) and PvDBPII (**b**) in patients with acute *P. vivax* infection (n = 29) compared with healthy controls (n = 18). The stability of IgG1 and IgG3 antibody responses to PvGAMA was shown from cross-sectional study (**c**) and the monitoring in individuals (**d**) of acute infected patients (n = 5). *Each symbol* represents the IgG1, IgG2, IgG3 and IgG4 titre of one individual. The *solid line* shows the median antibody titre in each group. The *dotted line* indicates the cut-off values

The patterns of IgG class-switching are influenced by the potential of antigen steering in the immune reaction, including the route of the antigen for entering the body and the chemical composition of the antigen. In this study, IgG1 and IgG3 were the predominant isotype responses to PvGAMA. IgG1 and IgG3 responses were significantly higher in patients who were acutely infected with *P. vivax* than in healthy controls (Fig. 4a). Of the most acutely infected patients, 48.7 and 64.1% produced anti-PvGAMA IgG1 and anti-PvGAMA IgG3 in response to PvGAMA. The comparing the anti-PvGAMA IgG isotype responses to PvGAMA and PvDBPII revealed differential responses to these antigens. The IgG2 subclass was the major isotype produced in response to the PvDBPII antigen (Fig. 4b). As there was a maintenance of total IgG specific to PvGAMA post-infection, the monitoring of anti-PvGAMA IgG1 and anti-PvGAMA IgG3 in cross-sectional study showed that IgG1 and IgG3 responses started to decrease significantly at 3 months post-infection and tended to decrease continuously at 9 and 12 months (Fig. 4c). However, an analysis of anti-IgG3 PvGAMA antibody titre in individual level showed that 3 from 5 seropositive patients maintained IgG3 responses up to 12 months after anti-malarial treatment whereas anti-IgG1 PvGAMA antibody titre in all patients was not stable (Fig. 4d).

### Cellular immune responses to PvGAMA

**Fig. 5:**
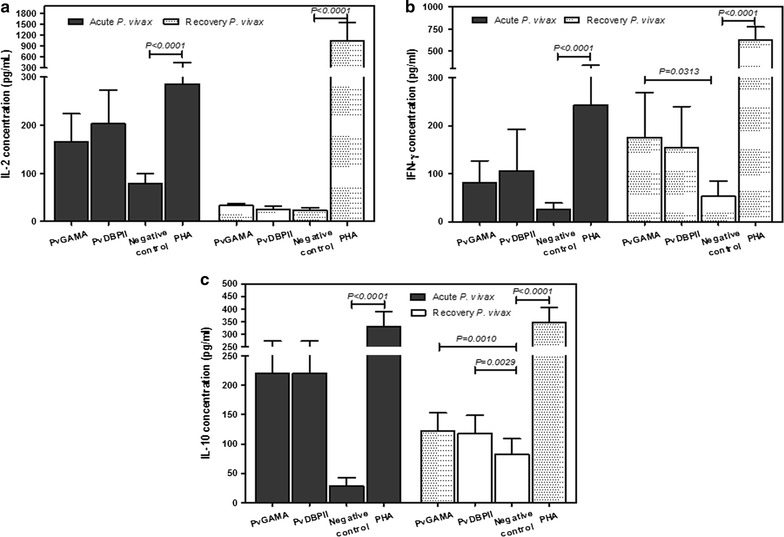
The cellular immune response to PvGAMA and PvDBP. PBMCs from patients with acute *P. vivax* infection (n = 11) and patients who had been in recovery from infection for 8–10 weeks (n = 11) were stimulated with recombinant PvGAMA or PvDBP in in vitro cultures for 5 days. The levels of **a** IL-2, **b** IFN-γ and **c** IL-10 were measured from cell culture supernatants upon rPvGAMA or rPvDBPII stimulation. *Bars* represent the median cytokine level of the study subjects

The differentiation of B cells can be triggered by two signaling pathways: namely, direct B cell triggering by the antigen itself or secondary signals from armed helper T cells. In this study, cytokine production was measured upon lymphocyte stimulation of in vitro cultures with PvGAMA or PvDBP. In *P. vivax*-infected PBMC’ cultures with rPVGAMA antigen, this antigen stimulated PBMCs to produce the cytokines IL-2 (PvGAMA = 166.0 ± 129.3 pg/mL; negative control = 78.1 ± 47.6 pg/mL; PHA = 285.64 ± 362.29 pg/mL), IFN-γ (PvGAMA = 80.5 ± 102.8 pg/mL; negative control = 25.9 ± 29.3 pg/mL; PHA = 242.91 ± 205.33 pg/mL) and IL-10 (PvGAMA = 220.7 ± 90.0 pg/mL; negative control = 28.5 ± 24.8 pg/mL; PHA = 328.57 ± 105.52 pg/mL); the cytokine levels were higher in antigen-stimulated cultures than in unstimulated control cultures (Fig. 5a–c). The ability of rPvGAMA-stimulated PBMCs to produce IL-2, IFN-γ and IL-10 was not significantly different from that of PBMCs stimulated with the recombinant PvDBPII antigen. PvGAMA-stimulated PBMC cultures from subjects who were in recovery from *P. vivax* infection showed significantly increased production of the cytokines IFN-γ (PvGAMA = 175.3 ± 229.1 pg/mL; negative control = 52.5 ± 79.7 pg/mL; PHA = 619.48 ± 351.53 pg/mL) and IL-10 (PvGAMA = 122.6 ± 99.9 pg/mL; negative control = 82.0 ± 89.1 pg/mL; PHA = 346.27 ± 194.04 pg/mL) (Fig. 5b, c).

## Discussion

Since *P. vivax* infection is not benign, as was considered previously, a vaccine against *P. vivax* is urgently needed in order to control and eradicate the disease. Because the *P. vivax* parasite has the unique ability to be reactivated from long-lasting, dormant liver stages called hypnozoites, the emerging of hypnozoites contribute to up to 80% of all *P. vivax* blood-stage infections and are responsible for the severity of the clinical symptoms of vivax malaria [16]. GPI-anchored antigens such as PvMSP-1, PvMSP-3, PvMSP-4, PvMSP-8, PvMSP-9, PvMSP-10, Pv12, Pv34, Pv38 and PvMSP1P have been proposed as vaccine candidates as they have the ability to trigger the immune response after natural *P. vivax* exposure and in vaccine-induced animal models [15, 17–25]. PvGAMA is an adhesion molecule required for *P. vivax* to invade Duffy-positive and Duffy-negative human erythrocytes [13]. An in vitro study using an erythrocyte binding inhibition assay showed that antisera against PvGAMA prevented the binding of PvGAMA to erythrocytes. In this study, the immunogenicity of PvGAMA as vaccine candidate was demonstrated in infected patients and after anti-malarial treatment. The results indicate that PvGAMA antigen can elicit an immune response and a positive anti-PvGAMA response was maintained at post-infection.

Antibodies are one key component of naturally acquired protective immunity against blood-stage parasites. The specific antibodies function by inhibiting merozoite invasion into new red blood cells, by enhancing the merozoite susceptibility to phagocytosis and by blocking the adherence of infected red blood cells to endothelial cells [26]. High levels of antigenicity of PvGAMA in *P. vivax* infection were observed. The seroprevalence of anti-PvGAMA IgG responses were 95.0–57.1% from acute phase through 3–12 months post-infection, whereas the villagers who lived in malaria-endemic areas for at least 5 years did not produce anti-PvGAMA responses. This result is consistent with the recent study by Cheng et al. [13] that reported a strong IgG prevalence of PvGAMA-ectodomain in *P. vivax*-infected patients from Korea, Thailand, Myanmar and China. Among 7 regions of PvGAMA-ectodomain, the F2 and F7 regions were the strongest binding activity to erythrocytes [13]. However, the antibody responses against each PvGAMA region to prevent the binding to human erythrocytes as well as the target epitope of anti-PvGAMA inhibitory antibodies will be required for further study.

Although it is widely believed that anti-malarial antibodies may not be long-lived in the absence of re-exposure, the maintenance of antibody responses to malaria has been debated by several previous studies [27, 28]. Some studies in animal models have shown that MSP1-19-specific IgG-producing memory B cells and antibody-secreting cells are detectable for up to 8 months following a primary infection [29]. Upon re-infection, there is an enhanced B cell response, and these cells rapidly form a germinal centre and generate a large population of long-lived plasma cells [30]. In humans, several studies have demonstrated the longevity of antibody responses to the malaria antigen in an area of low transmission; both antibody and memory B cell responses to malaria antigens were found to be stably maintained over time [28, 31]. By contrast, studies carried out in areas of high malaria transmission have shown short-lived antibody responses to blood-stage antigens [32–35]. However, there is no correlation between the level of malaria-specific antibody and memory B cells, since long-lasting *Plasmodium*-specific memory B cells were observed in exposed individuals who had short-lived antibody titres [27]. Here, the naturally acquired anti-PvGAMA response was observed in individuals during *P. vivax* infection and at 3, 9 and 12 months post-infection. The antibody response was greatly reduced at 3 months post-infection, and approximately 54% of the patients could maintain antibody responses, whereas 46% had become seronegative 3 months post-infection. Interestingly, anti-PvGAMA responses in some patients were appropriately maintained at seropositive levels at 9 months and 12 months in the absence of re-infection as the nested PCR of all recovery samples was shown negative at all period of monitoring. However, the anti-PvGAMA longevity by means of a cohort study that observed the association between clinical disease and maintenance of antibody responses in natural *P. vivax* exposure, as previously reported in other malaria antigens [36–38] will be useful for the development of an anti-malarial vaccine.

Infection with a pathogen or immunization with a vaccine induces the production of antibodies that provide protection against the pathogen. The examination of the antibody isotypes produced by individuals in response to infection can help to characterize the immune responses to that specific pathogen. Each IgG isotype has a unique effector function in the context of an immune response. The Fc portion of the cytophilic isotypes IgG1 and IgG3 mediates effector functions such as antibody-dependent cellular cytotoxicity (ADCC) and complement-dependent cytotoxicity (CDC) by co-operating with monocytes, via FcγRI and FcγRII receptors. Conversely, levels of the non-cytophilic isotypes IgG2 and IgG4 have been reported to be negatively correlated with anti-malarial protection [39]. In the current study, IgG1 and IgG3 were the predominant isotypes produced in response to PvGAMA antigen. The IgG3 response was stable, as indicated by the maintenance of the seropositivity during infection and post-infection in some seropositive individuals. Thus, the ability of patients maintained anti-IgG3 PvGAMA antibodies may protect clinical malaria as from previous studies had been shown the association between anti-IgG3 PfMSP1 antibodies and the prolonged period without malaria infection [18].

The collaboration between CD4^+^ T cells and B cells via the ligation of co-stimulatory molecules expressed on the surfaces of activated T cells is responsible for humoral immunity against most protein antigens. Two major subsets of CD4^+^ T cells, T helper cell 1 (Th1) and Th2 cells can promote different patterns of cytokine production, which result in distinct immune responses. In malaria disease, a balance between the Th1 and Th2 cytokines is critically required for determining parasitaemia or clinical outcome [26, 40, 41]. A protective role of IFN-γ against the blood stage of malaria had been shown in protected individuals living in malaria endemic areas [42, 43]. In this study, PvGAMA antigen-stimulated PBMCs from *P. vivax*-infected subjects were shown to produce a high-level of the cytokine IFN-γ. The induction of the IFN-γ in response to PvGAMA antigen may function by enhancing the production of PvGAMA-specific cytophilic IgG1 and IgG3 antibodies as the antibody responses of these two antibody subtypes increased during infection. The elevation of IFN-γ upon PvGAMA stimulation could help switching of IgG subclasses biased toward IgG1 and IgG3 responses to *P. vivax* parasites [44]. Moreover, the cytokine IL-10 was also detected in both PvDBP-stimulated and PvGAMA-stimulated PBMC culture supernatants. The elevation of IL-10 levels upon the re-stimulation with PvGAMA in vitro explained the development of IL-10 producing cell during *P. vivax* infection and these cells activated after re-stimulation with specific antigen. However, in this study, the IL-10-producing cells responded to PvGAMA were not identified. It is possible that source of IL-10 can be FOXP3^+^CD4^+^CD25^+^ T cells (Treg) as they were significantly expanded in acute infected *P. falciparum* and *P. vivax* patients [45, 46]. However, the function of IL-10 in malaria is still unclear as IL-10 producing CD4^+^ T cells were involved in dampening severe pathology during *Plasmodium* infection [45, 47, 48] whereas, it had also been reported to reduce levels of IFN-γ and TNF-α, which helps the parasites to escape the immune response [49]. In addition, IL-10 cytokine from PvGAMA stimulation may assist B cells in antibody production, as a previous study found that *Plasmodium* antigen-specific production of IL-10 was related to an antibody response [50].

## Conclusions

In this study, the immunogenicity of the PvGAMA antigen was demonstrated during acute phase and post-infection with *P. vivax*. The high seroprevalence of PvGAMA, approximately 95%, was found in patients who were acutely infected with *P. vivax*. The monitoring of antibody responses to PvGAMA demonstrated that the antibody titre was appropriately maintained at a positive titre after anti-malarial treatment in the absence of reinfection. IgG3 isotype antibody was the major antibody produced in response to the PvGAMA antigen. The elevation of IFN-γ upon re-stimulation with PvGAMA was considered for Th1 response and promoting of IgG3 subtype switching. An increased understanding of the immunogenicity of PvGAMA could help in the development of a vaccine against *P. vivax* infection.

## Abbreviations

PvGAMA: *Plasmodium vivax* glycosylphosphatidylinositol-anchored micronemal antigen
DARC: Duffy antigen receptor for chemokine
PBMCs: peripheral blood mononuclear cells
ELISA: enzyme-linked immunosorbent assay
LPA: lymphocyte proliferation assay
PHA: phytohemagglutinin
RPMI: Roswell Park Memorial Institute
TMB: tetramethylbenzidine
